# Electroacupuncture reverses endothelial cell death and promotes angiogenesis through the VEGF/Notch signaling pathway after focal cerebral ischemia‐reperfusion injury

**DOI:** 10.1002/brb3.2912

**Published:** 2023-02-14

**Authors:** Wenyu Zhang, Lin Han, Yan Wen, Lixian Su, Yibing Li, Xudong Luo

**Affiliations:** ^1^ Department of Acupuncture and Moxibustion First Teaching Hospital of Tianjin University of Traditional Chinese Medicine Tianjin China; ^2^ National Clinical Research Center for Chinese Medicine Acupuncture and Moxibustion Tianjin China; ^3^ Tianjin University of Traditional Chinese Medicine Tianjin China; ^4^ Department of Traditional Chinese Medicine Tianjin Medical University General Hospital Tianjin China; ^5^ Department of Information Science and Engineering Yunnan University Kunming, Yunnan Province China

**Keywords:** angiogenesis, cerebral ischemia‐reperfusion injury (CIRI), electroacupuncture (EA), endothelial cells (ECS), VEGF/Notch signaling

## Abstract

**Background:**

Angiogenesis is an important mechanism of recovery from ischemic stroke. Recent studies have found that there is a close relationship between the VEGF/Notch pathway and angiogenesis. It is unknown whether EA can exert a brain protection effect and promote angiogenesis by acting on the VEGF/Notch signaling pathway after focal cerebral ischemia‐reperfusion injury (CIRI).

**Methods:**

The Middle Cerebral Artery occlusion/Reperfusion (MCAo/R) model was established, in which rats were subjected to occlusion with ischemic intervention for 30 min, followed by reperfusion for 8 h, 1 day, 3 days, and 7 days. The first EA treatment was performed 90 min after the animal model was successfully established, and then EA treatments were performed once a day for 7 days. The 2,3,5‐triphenyltetrazolium chloride staining and neurological deficit examination were performed to assess the level of CIRI and neuroprotection by EA. Expression levels of VEGFA, Notch1, and Hes1 proteins were measured via western blotting, while the morphological changes of ECs and microvasculature in the cortex were determined using an ultrastructural observation method.

**Results:**

EA treatment of PC6, GV26, and SP6 can significantly improve the neurological function of MCAO/R rats, reduce the volume of cerebral infarction, and modulate the ultrastructure of ECs and microvessels in pathological states. Western blotting revealed that EA increased VEGFA protein expression at 8 h and 3 days after CIRI, as well as Notch1 protein expression at 1 and 7 days. Subsequently, EA activated the VEGF/Notch pathway, increasing the expression of the downstream target protein Hes1, reversing EC death, and promoting angiogenesis.

**Conclusion:**

Our findings showed that EA plays a role in promoting angiogenesis following focal CIRI, and we hypothesized that this was due to the regulation of ECs by the EA‐activated VEGF/Notch signaling pathway.

## Graphical abstract

Electroacupuncture treatment of PC6, GV26, and SP6 promotes VEGFA protein expression 8 h and 3 days after CIRI, as well as Notch1 protein expression 1 and 7 days. The VEGF/Notch pathway was then activated, raising Hes1 protein expression, reversing EC death, and promoting angiogenesis. (The figure “MCAo/R” is cited from https://www.medicalnewstoday.com/articles/324998 and the figure “Cerebral microvascular ECs” is cited from “https://www.cellapplications.com/endothelial”).

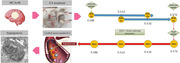

## 1 INTRODUCTION

Cerebral Ischemic Stroke (CIS) is the major cause of disability and death worldwide, putting human health at considerable risk (Virani et al., 2021). After CIS, the quick reestablishment of blood flow boosts the level of tissue oxygenation, resulting in the second burst of cell damage (Amani et al., 2019). This is called cerebral ischemia‐reperfusion injury (CIRI) (Sharma et al., 2020). Endothelial cells (ECs) are the primary constituents of the blood‐brain barrier, and their dysfunction and death contribute significantly to the pathological progression of CIRI (Li et al., 2020a,b; Zhang et al., 2021; Saberianpour et al., 2021; Nemcovsky et al., 2019; Nitzsche et al., 2021). Although CIRI is a complex pathological process involving multiple cytokines and signaling pathways, it is a deterioration of brain tissue that can be partially repaired through interventions (Khamchai et al., 2022; Tuo et al., 2022). Secondary brain injuries occur after focal CIRI. Due to the increasing brain edema, the blood‐brain barrier (BBB) is severely disrupted, leading to the infiltration of peripheral immune cells and exacerbating the inflammatory response of the central nervous system (CNS) (Kawabori & Yenari, 2015). Accumulating evidence shows that angiogenesis not only protects brain tissue (Zhang et al., 2000), but also contributes to neuronal recovery (Hatakeyama et al., 2020). It has been reported that in the rodent MCAO model, EC began to proliferate as early as 12−24 h after ischemia, but the cerebral microvessels in the core ischemic area and ischemic penumbra recombined within 4−7 days (Hayashi et al., 2003; Kanazawa et al., 2017). At the same time, neuronal remodeling and axonal growth occurred 14 days after the stroke (Kanazawa et al., 2017). Therefore, promoting angiogenesis may become a promising therapeutic strategy for the treatment of ischemic stroke.

Angiogenesis (AG) is an important process of neurovascular unit remodeling after ischemic stroke (Hatakeyama et al., 2020; Seet et al., 2012). It not only increases oxygen and nutrition supply to the ischemic penumbra (Zhang et al., 2002), but also regulates the growth of axons and neurogenesis (Liu et al., 2014; Widenfalk et al., 2003), which can protect brain tissue from cerebral ischemic damage. Previous studies have demonstrated that increased microvessel density in the peri‐infarct region contributes to prolonging the survival time of stroke patients, suggesting that angiogenesis is important for brain tissue repair after stroke (Krupinski et al., 1994). In addition, angiogenesis also plays a key role in the recovery of neurological function after ischemic stroke, as it facilitates the establishment of collateral circulation and increases the migration of oxygen, glucose, nutrients, and neural stem cells (NSCs) to the ischemic area (Beck & Plate, 2009; Ruan et al., 2015). It is considered one of the most promising strategies to treat cerebral infarction. However, the self‐repair ability of cerebral ischemic tissue is limited and cannot effectively improve the blood supply and neurological function recovery after cerebral ischemia. Therefore, it is very important for us to explore an effective method as soon as possible to restore the blood supply in the ischemic area, promote angiogenesis, and help the damaged brain tissue and neurological function recover in time.

VEGF and Notch signaling pathways are important parts of many signaling pathways that are related to angiogenesis after cerebral ischemia (Liang et al., 2020, 2021). VEGF (vascular endothelial growth factor) is a specific mitogen for ECs and a potent regulator of angiogenesis, capable of initiating a complex cascade of events including EC activation, assembly of new vascular structures, mural cell recruitment, and vascular stabilization (Gianni‐Barrera et al., 2011). Lobov et al. (2007) found that VEGF is located upstream of the Notch signaling pathway. Studies have shown that there is a complex coregulatory relationship between VEGF and Notch pathways (Jin et al., 2014). After VEGF binds to related receptors, it can activate the Notch signaling pathway by increasing the expression level of Notch ligands such as DLL4. This indicates that the regulation of angiogenesis by the Notch signaling pathway is accompanied by the close participation of VEGF. The Notch pathway is mainly composed of Notch receptors, ligands, and target genes, which determine the differentiation of cells and the occurrence of tissues. When the ligand binds to the receptor, the Notch molecule is activated, releasing Notch1 (NICD) and initiating its downstream transcriptional targets, such as Hes1, to play a regulatory role. Some researchers used the immunofluorescence double staining method to observe that the expression of NICD in vascular ECs in the ischemic cortex was significantly upregulated after 3 days of cerebral ischemia compared with the control group (Lobov et al., 2007). It was still expressed at a high level after 3 days, indicating that Notch1 may participate in the regulation of angiogenesis after cerebral ischemia by upregulating its target gene, the Hes1 protein. Even though the articles on the VEGF‐Notch signaling pathway and angiogenesis have clues that can be looked into, most studies go into detail about the role of a certain protein in one of the signaling pathways, such as a single study on the role of the VEGF pathway in angiogenesis after CIRI or a single study on the role of the Notch pathway in angiogenesis after CIRI. However, based on previous evidence, there is a complete link between VEGF and Notch signaling pathways. The innovation of our research is to focus on the cascade reaction of the expression of angiogenic factors in the VEGF/Notch pathway caused by CIRI, including the dynamic changes of angiogenesis in the upstream, middle, and downstream target proteins of this pathway. We are dedicated to investigating the molecular mechanism of EA intervention on the dynamic expression of VEGF/Notch pathway angiogenic factors and precise regulation of angiogenesis as they relate to morphological changes in ECs, thus revealing the brain protective effect of EA.

Electroacupuncture (EA) can influence cell electrophysiology and has a high therapeutic potential for angiogenesis, neurogenesis, and cerebral circulation reconstruction in the ischemic area following a stroke (Wang et al., 2021). Furthermore, EA can also affect certain signaling pathways and play a role in reducing cerebral edema and antiapoptosis after focal CIRI (Long et al., 2021; Zou et al., 2015). The role of EA in promoting angiogenesis after cerebral ischemia is clear, but its ability to regulate the VEGF/Notch signaling pathway is still limited. However, it is worth noting that the latest evidence shows that EA can activate the HIF‐1/VEGF/Notch1 signaling pathway and promote angiogenesis after ischemic stroke through exon miR‐210 (Xu et al., 2022). This study aims to analyze the particular mechanism of EA as an intervention method for supporting angiogenesis at various time periods after CIRI and to offer a scientific basis for the clinical treatment of cerebral ischemia disorders.

## 2 MATERIALS AND METHODS

### 2.1 Laboratory animals and groups

A total of 128 eight‐week‐old male Wistar rats (SPF grade), weighing (200 ± 20) g, were provided by Beijing Vital River Laboratory Animal Technology Co., Ltd., laboratory animal certificate number: SCXK (Beijing) 2016‐0011. The experimental animals were kept at room temperature (20–25°C), light time (07:00–19:00), eating and drinking freely. According to the random number table, the experimental animals were divided randomly into four groups: the blank group (*n* = 8), the sham surgery group (*n* = 8), the MCAo/R model group (*n* = 56), and the EA group (*n* = 56). The latter two groups were divided into four subgroups at 8 h, 1 day, 3 days, and 7 days after ischemia reperfusion, with 14 rats in each group. Eight rats in each group were used for the neurological deficit score, of which five were used to detect cerebral infarction volume and three were used for ultrastructural electron microscopic observation. The other six were used to detect the VEGF/Notch signaling pathway (VEGFA, Notch1, and Hes1 protein). All experiments were performed following the National Institutes of Health Laboratory Animal Care and Use Guidelines (NIH Publications No. 80‐23) and were approved by the Animal Ethics Committee of Tianjin University of Traditional Chinese Medicine, China (Approval No. TCM‐LAEC2019018).

### 2.2 Model preparation

The middle cerebral artery occlusion/reperfusion (MCAo/R) model was made according to Zea‐Longa's suture method (Longa et al., 1989). The rats were fasted for 12 h, weighed, and anesthetized by intraperitoneal injection of pentobarbital (Merck Co., Ltd., Beijing, China; 50 mg/kg, i.p.). Rats were fixed on the surgical plate in the dorsal position. After skin preparation and disinfection, make the left incision 1.5−2 cm from the neck's middle. The subcutaneous fascia was bluntly dissected to expose the left sternocleidomastoid‐sternohyoid triangle. The left common carotid artery (CCA) and external carotid artery (ECA) were bluntly separated, the ECA was ligated, and the distal and proximal ends of the CCA bifurcation were clamped with artery clamps, respectively. After puncturing the proximal blood vessel wall with a 1 mL syringe needle, insert the nylon filament (Beijing Sunbio Biotech Co., Ltd., Beijing, China) with a diameter of 0.260 mm slowly along the needle hole (the filament tip is polished round and smooth with fine sandpaper). When the filament reaches the bifurcation, release the artery clamp and continue sending the filament into the internal carotid artery (ICA) until slight resistance is encountered. The depth of the filament into the ICA is 18–20 mm. At this time, the filament relies on its front end to stop the blood from flowing back through the anterior cerebral artery (ACA). The filament blocks the ICA and the posterior communicating artery, blocking the blood supply of the MCA on one side. After successful filament insertion, ligate the CCA and the pinhole, release another artery clamp, suture in layers, block the blood flow for 0.5 h, remove the filament, and realize reperfusion through the contralateral cerebral communicating artery. The other steps in the sham surgery group were the same as those in the MCAo/R group, except that the nylon filament was not inserted. After waking up, animals with scores of 1, 2, and 3 on the Longa 5‐point method (Longa et al., 1989) were chosen as experimental subjects.

### 2.3 Electroacupuncture treatment

We kept the rats awake during the EA treatment. All rats wearing rat clothes were fixed on the table with adhesive tape. According to “Experimental Acupuncture Science” (Guo, 2016), “Shuigou” (GV26), “Neiguan” (PC6), and “Sanyinjiao” (SP6) were selected. GV26 was located in the middle of the cleft lip, 1 mm below the tip of the nose. PC6 was located 3 mm above the wrist joint on the medial side of the forelimb between the ulna and the radius and was punctured vertically to a depth of 1 mm; SP6 was located 1 cm above the tip of the medial malleolus of the hind limb. Each acupoint was needled for 20 min. During the needle retention period, the filiform needle handles at PC6 and SP6 acupoints on the affected side were respectively connected to the HANS nerve acupoint stimulator, and the EA waveform was set as a distant dense wave. The frequency was 2/15 Hz (adjusted to the muscle twitch threshold), and the current was 1 mA. The first EA treatment was performed 90 min after the animal model was successfully established, and then EA treatments were performed once a day for seven days. The rats in the blank group, sham surgery group, and MCAo/R model group received the same grasping but were not given EA treatment. The length of the acupuncture needles (Huatuo, Suzhou Medical Products Co., Ltd., Suzhou, China) is 40 mm, and the diameter is 0.30 mm. The HANS nerve acupoint stimulator HANS‐200E (Nanjing Jisheng Medical Technology Co., Ltd., Nanjing, China) was used as the EA instrument. The product standard number is YZB/Su 0049–2008.

### 2.4 Effect index and measurement method

#### 2.4.1 Neurological deficit score in MCAo/R rats

**FIGURE 1:**
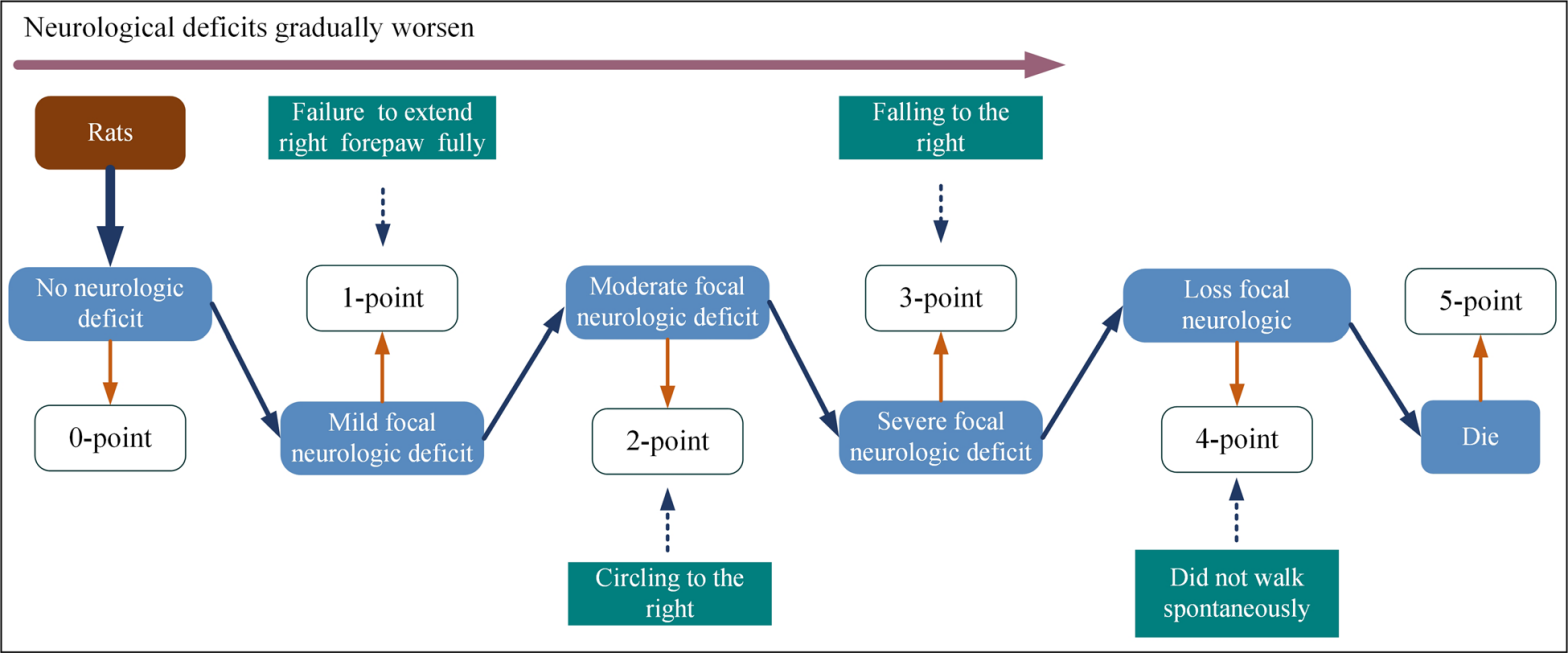
Zea‐Longa 5‐point method.

The neurological function was assessed by professional staff who did not participate in MCAo/R surgical and EA treatment in accordance with the Zea‐Longa 5‐point method (Longa et al., 1989) at 8 h, 1 day, 3 days, and 7 days after MCAo/R surgery. The higher the score, the more serious the neurological deficit. The specific methods and scoring standards are shown in the figure below (Figure 1).

#### 2.4.2 2,3,5‐Triphenyltetrazolium chloride (TTC) staining

The volume of cerebral infarction in rats was detected by using the TTC staining method (Cheng et al., 2015). Place the brain tissue in the refrigerator at −20°C for 15 min until the brain tissue becomes hard. Slice the brain from the frontal to occipital poles to make coronal slices. Each slice is about 2 mm thick and cut into six pieces. Put the brain slices in 1% TTC phosphate buffer (pH = 7.4) (Nasi Biotechnology Co., Ltd., Shanghai, China) and cover them with tin foil. Place them in a 37°C incubator for 10−15 min. Turn the brain slices with a brush to ensure even staining. TTC reacts with dehydrogenase in normal tissues and turns red, but in ischemic tissues, dehydrogenase activity decreases and fails to respond, so cerebral infarct tissue appears pale. Use Image‐Pro Plus software (version 6.0, Media Cybernetics, Rockville, MD, USA) to calculate cerebral infarction volume. Infarct volume (%) = [(contralateral hemisphere volume‐normal volume of the infarct side) ÷ contralateral hemisphere volume] × 100%.

#### 2.4.3 Western blot

Six rats per group were used for western blot detection at the corresponding sampling time. Before protein cleavage, PMSF (Sangon Biotech) was added to RIPA Lysis Buffer I (Sangon Biotech), lysed on ice for 1 h, and the supernatant was collected. After that, the protein concentration was measured by the BCA method. Absorb 50 g of protein per sample's concentration, mix with loading buffer in a 4:1 ratio, and dilute to 20 L. Fill it up with ddH2O. The concentrated gel and separation gel electrophoresis voltages are 80 V and 100 V. Electrophoresis is complete when bromophenol blue reaches the bottom of the gel. The PVDF membrane was blocked in 5% skim milk for 2 h after the protein sample was transferred. After blocking, the membrane was gently washed with TBST, rabbit anti‐VEGFA (1:100, Santa Cruz Biotechnology), mouse anti‐Notch1 (1:100, Santa Cruz Biotechnology), and rabbit anti‐Hes1 (1:100, Santa Cruz Biotechnology). Antibodies were incubated overnight at 4°C. After 16 h, the membrane was taken out and washed on the TBST shaker for 3−5 min. After washing, add anti‐HRP goat anti‐mouse IgG‐Fc II (Cell Signaling Technology) and shake for 1 h. Darkroom imaging with ECL color developing solution. ImageJ software was used to measure the gray value of VEGFA, Notch1, Hes1, and ‐actin proteins. Finally, the final relative expression was compared according to the ratio.

#### 2.4.4 Ultrastructure observation of vascular ECs

Three rats in each group that met the inclusion criteria were anesthetized according to the corresponding sampling time. The left cerebral cortex was separated, and the tissue mass in the ischemic focus area (left parietal and temporal cortex tissue) was cut into 1 mm × 1 mm × 1 mm and fixed in 2.5% glutaraldehyde solution (Huifengda Chemical Co., Ltd., Jinan, China) at 4°C. The fixed brain tissue was rinsed, dehydrated, impregnated, and embedded in epoxy resin. The prepared brain tissue was repositioned under the light microscope and ultrathinly sectioned. The thickness of each piece of brain tissue was 50 nm. Remove the slices from the copper net for drying, and double stain with 3% lead citrate (Suihua Biological Technology Co., Ltd., Jinan, China) + 0.1% sodium acetate (Macleans Biochemical Technology Co., Ltd., Shanghai, China). Cell morphology and ultrastructure changes on the slices were observed under the Hitachi transmission electron microscope (H600; Hitachi, Tokyo, Japan). The photographs were taken with a Nikon digital camera. (D850; Nikon, Tokyo, Japan).

### 2.5 Statistical analysis

The data are analyzed by SPSS 18.0 system software (SPSS, Chicago, IL, USA), and the inspection level is (α = 0.05). All data are tested by one sample Kolmogorov–Smirnov test (K–S test). For those with a normal distribution (*p* > .05), the statistical description is expressed by mean ± SD, and the homogeneity of variance test is performed. One‐way ANOVA is used for multigroup comparison of the normal distribution data. The least significant difference method (LSD test) was used for the pairwise comparison of each experimental group. A nonparametric Kruskal–Wallis test is used for nonnormal distribution data. *p* < .05 is statistically significant.

## 3 RESULTS

### 3.1 Focal CIRI caused edema, necrosis, and apoptosis in MCAo/R ECs

**FIGURE 2:**
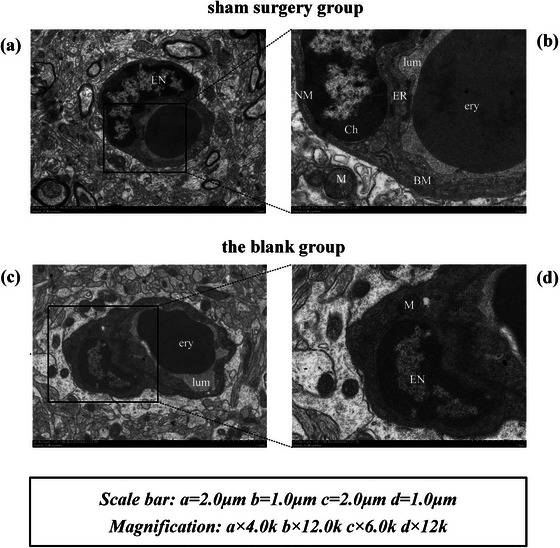
Ultrastructure observation of sham surgery group and blank group. The figure shows two microvessels with normal structure, large lumen, and regular shape. Areas b and d are two detailed views of areas a and c. Two arch‐shaped ECs can be seen on the upper right side of the microvessels. *Note*: ery (erythrocyte); PC (pericyte); EN (Endothelial Nucleus); BM (Basement Membrane); V (vacuole); G (Golgi apparatus); lum (lumen); NM (Nuclear Membranes); Ly (Lysosome); M (Mitochondria); ER (Endoplasmic Reticulum); PMV (Plasma Membrane Vesicles); EC (Endothelial Cell).

**FIGURE 3:**
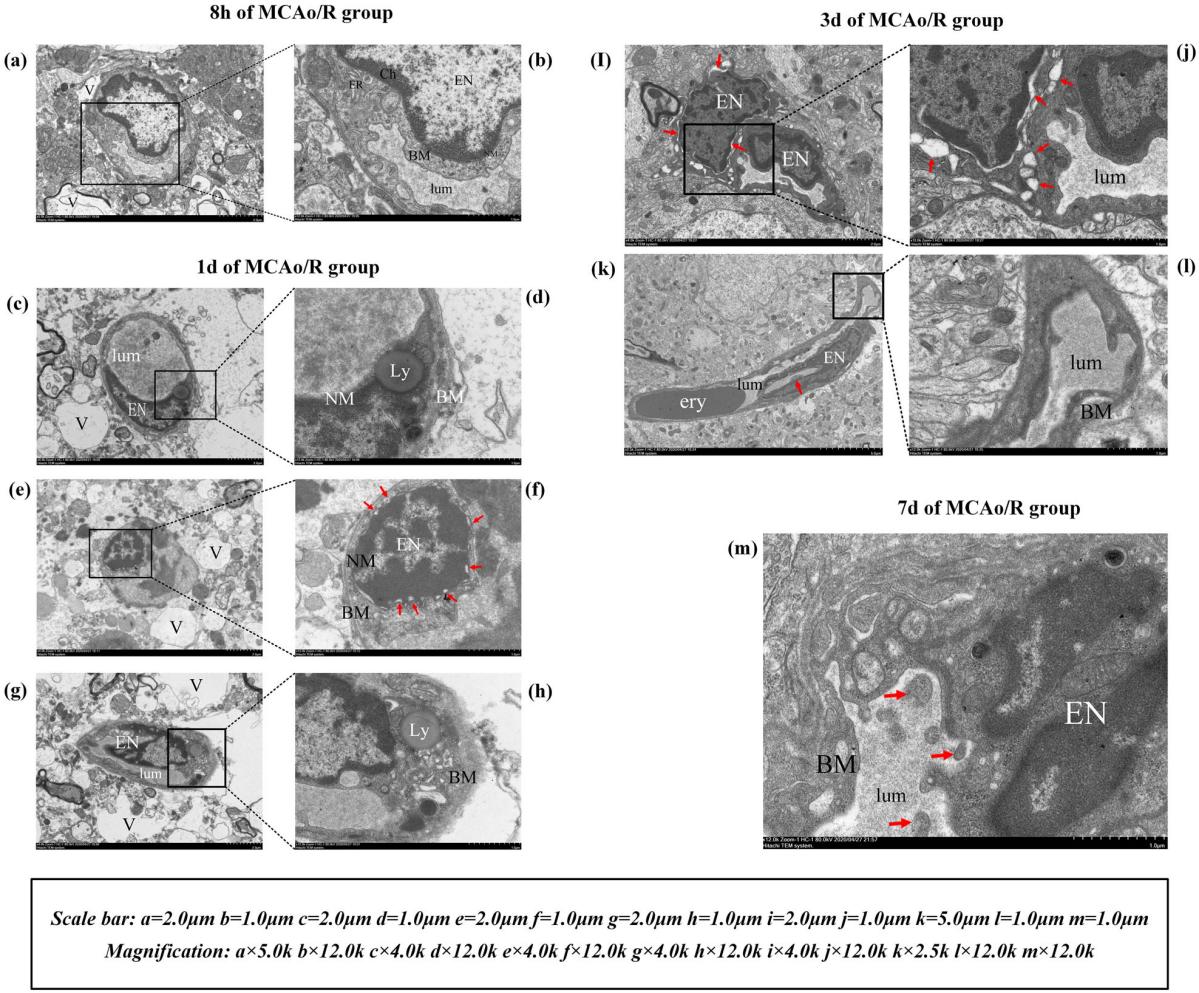
Ultrastructure observation of MCAo/R group.

**FIGURE 4:**
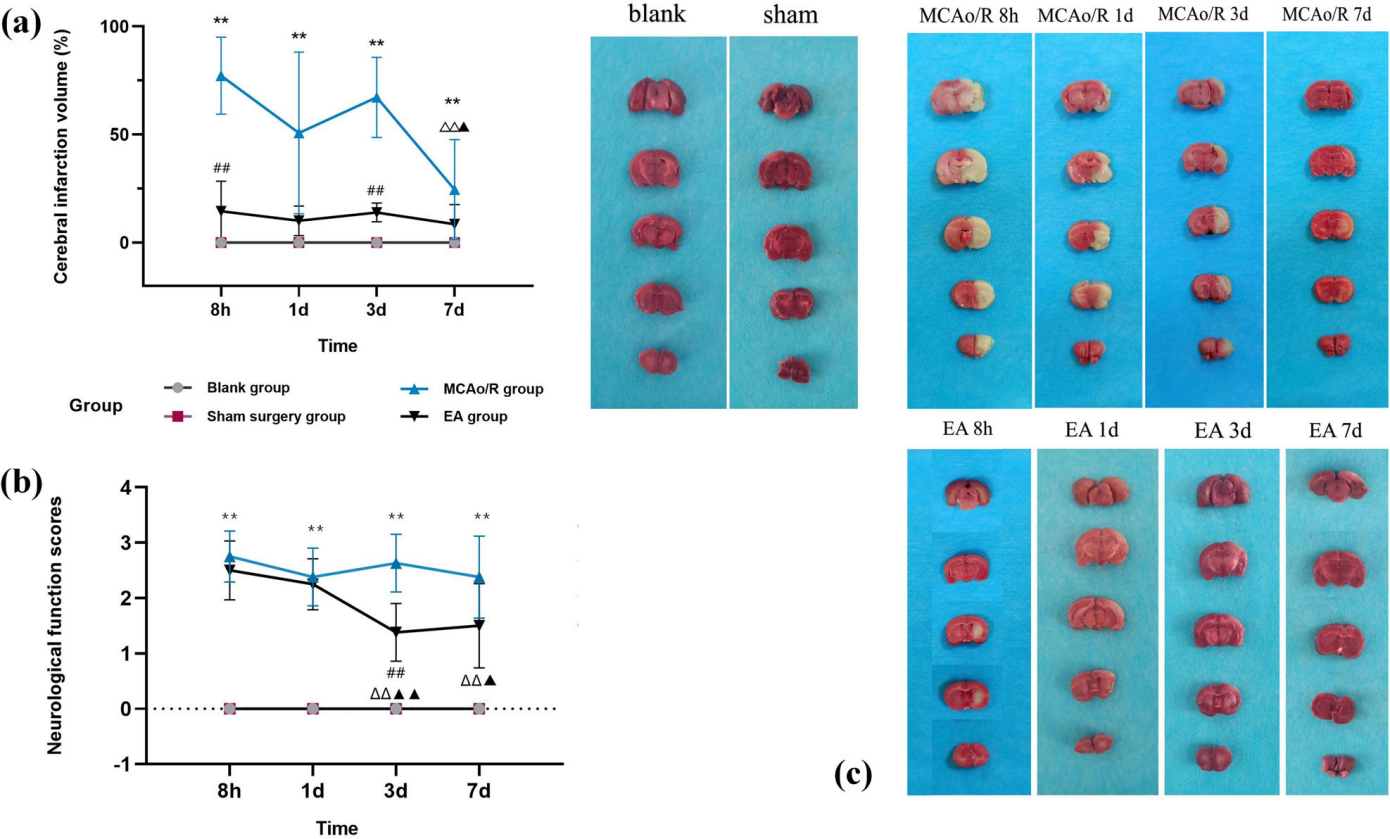
Changes of the cerebral infarction volume (%) and neurological function scores in each group of rats at different times. (a) Quantitative results of the cerebral infarction volume. Data are presented as the mean ± SD (*n* = 5 rats). Compared with the sham surgery group at the same time, ^**^ *p* < .01. Compared with the MCAo/R group at the same time, ^##^ *p* < .01. Compared with 8 h in the MCAo/R group, ^ΔΔ^ *P* < 0.01, compared with 3 days in the MCAo/R group, ^▲^ *p* < .05. (b) Quantitative results of Zea‐Longa neurological function scores (*n* = 8 rats). Compared with the sham surgery group at the same time, **p < .01; compared with the MCAo/R group at the same time, ^##^ *p* < .01; compared with 8 h after MCAo/R surgery in the EA group, ^ΔΔ^ *p* < .01, compared with 1 day after MCAo/R surgery in the EA group, ^▲^ *p* < .05, ^▲▲^ *p* < .01. (c) The cerebral infarction areas were measured by 2,3,5‐triphenyltetrazolium chloride staining. The white represents the ischemic area, and the red shows the normal area.

After MCAo/R surgery, the infarct volume of the MCAo/R rats expanded in 8 h, 1 day, 3 days, and 7 days (all *p* < .01), indicating that the model was successful (Figure 4a and c). Then we analyzed the ECs pathological changes of MCAo/R rats from the perspective of ultrastructural analysis. The left cerebral cortex microvessels and ECs of rats in the sham surgery group and blank group have complete structure and normal morphology. The microvessels and surrounding tissues are healthy. Vascular ECs maintain homeostatic function (Figure 2). Eight hours after focal CIRI, the ultrastructure showed the characteristics of the initial phase of vascular EC necrosis: cell swelling (Figure 3a and b). One day after focal CIRI, more vacuoles appear around ECs, organelles are swollen, endothelial nuclei are fragmented, the nucleus membrane is no longer intact, and lysosomes increase. Cell edema is more intense in this phase than in the initial stages, and early vascular EC necrosis may occur (Figure 3c–h). The obvious transition occurred 3 days after the focal CIRI. The ultrastructure showed the characteristics of ECs in preapoptosis: A deepening of nucleus chromatin accompanies the nucleus shrinkage of ECs. There are multiple vacuoles of different sizes around ECs—the formation of plasma membrane blebs, and the nucleus membrane is sunken inward. The inside of the nucleus is fragmented, but the nucleus membrane is still intact—a typical feature before cell apoptosis. Cell edema at this stage is not the primary manifestation (Figure 3i–l). Notably, 7 days after focal CIRI, the ultrastructure displayed signs of the last stage of EC apoptosis, including endothelial nucleus fragmentation and nucleus dissolution. The nucleus membrane disappearance, organelle damage, and apoptotic cell debris fall off into the lumen (Figure 3m).

### 3.2 The cerebral infarction volume of MCAo/R rats changed, the expression of Hes1 protein dropped, and the neurological function deteriorated

**FIGURE 5:**
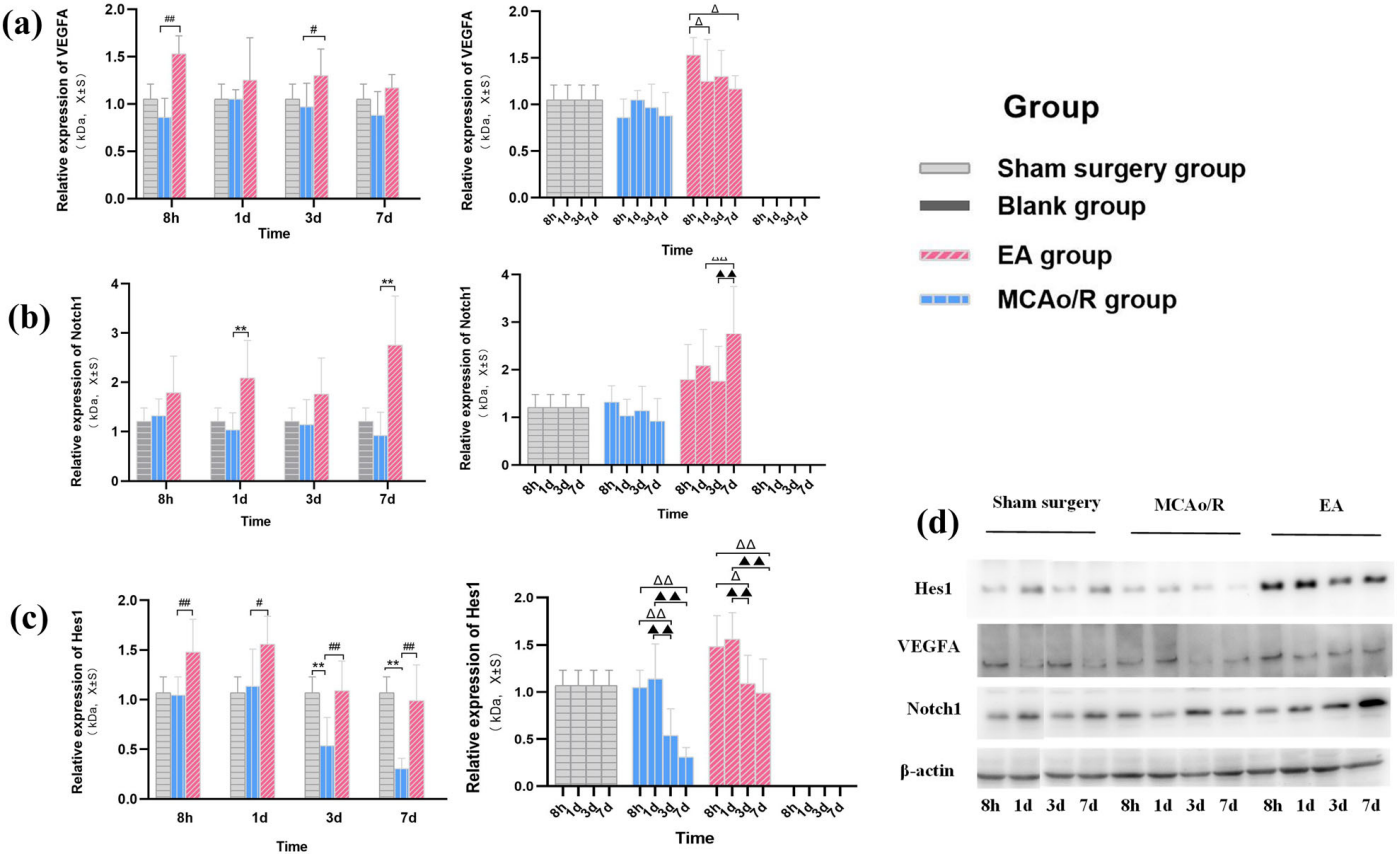
Effect of EA on the expression of VEGFA, Notch1, and Hes1 in MCAo/R rats. (a and b) Comparison of VEGFA protein expression in different groups of rats (VEGFA/β‐actin) (*n* = 6). (a) Compared with the MCAo/R group at the same time, ^#^ *p* < .05, ^##^ *p* < .01. (b) Compared with 8 h in the EA group, ^Δ^ *P* < 0.05. (c and d) Comparison of Notch1 protein expression in different groups of rats (Notch1/β‐actin) (*n* = 6). (c) Compared with the MCAo/R group at the same time, ^##^ *p* < .01. (d) Compared with 8 h in the EA group, ^ΔΔ^ *P* < 0.01, compared with 3 days in the EA group, ^▲▲^ *p* < .01. (e and f) Comparison of Hes1 protein expression in different groups of rats(Hes1/β‐actin)(*n* = 6). (e) Compared with the sham surgery group at the same time, ^**^ *p* < .01. Compared with the MCAo/R group at the same time, ^#^ *p* < .05, ^##^ *p* < .01. (f) Compared with 8 h in the MCAo/R group and EA group, ^ΔΔ^ *p* < 0.01, ^Δ^ *p* < 0.05, compared with 1 day in the MCAo/R group and EA group, ^▲▲^ *p* < .01. (g) Representative western blots of Hes1, Notch1, and VEGFA in each group. Data are presented as mean ± SD.

We continued to detect the cerebral infarction volume changes at 8 h, 1 day, 3 days, and 7 days after focal CIRI in rats and found that they were related to the pathological changes of ECs. As shown in Figure 4a, the volume of cerebral infarction 7 days after MCAo/R surgery was significantly lower than it was 8 h and 3 days later (*p* < .05 and *p* < .01), suggesting that as the infarction time continues to prolong, rats show a certain degree of self‐regulation ability. The ECs develop from severe swelling and necrosis at 8 h and 1 day in the early stage to shrinkage and apoptosis of the ECs at 3 days and 7 days. Brain edema will gradually decrease under the self‐regulation of brain tissue ECs, and the infarct volume will also shrink accordingly (Figure 4b), which shows a parallel relationship with the formation and resolution of tissue edema after focal CIRI. In addition, the expression level of Hes1 protein in the MCAo/R group peaked at 1 day after focal CIRI, and showed a downward trend in the subsequent 3 days and 7 days. Compared with 8 h and 1 day in the MCAo/R group, the expression of Hes1 protein decreased at 3 and 7 days (*p* < .01) (Figure 5f). The loss of Hes1 protein 3 and 7 days after focal CIRI (Figure 5e) may lead to severe defects of brain cells, which are closely related to EC apoptosis (Figure 3i–m) and affect the neurological function of rats. At the same time, compared with the sham surgery group, the neurological deficit scores of the model group were significantly increased at each time point (all *p* < .01) (Figure 4b), this suggests that with the destruction of ECs in different periods, the expression of Hes1 protein decreased gradually, MCAo/R rats’ neurological function is gradually lost.

### 3.3 EA activated the VEGF/Notch pathway, regulated EC lesions, relieved brain edema 8 h and 1 day after CIRI, and reduced cerebral infarction volume

**FIGURE 6:**
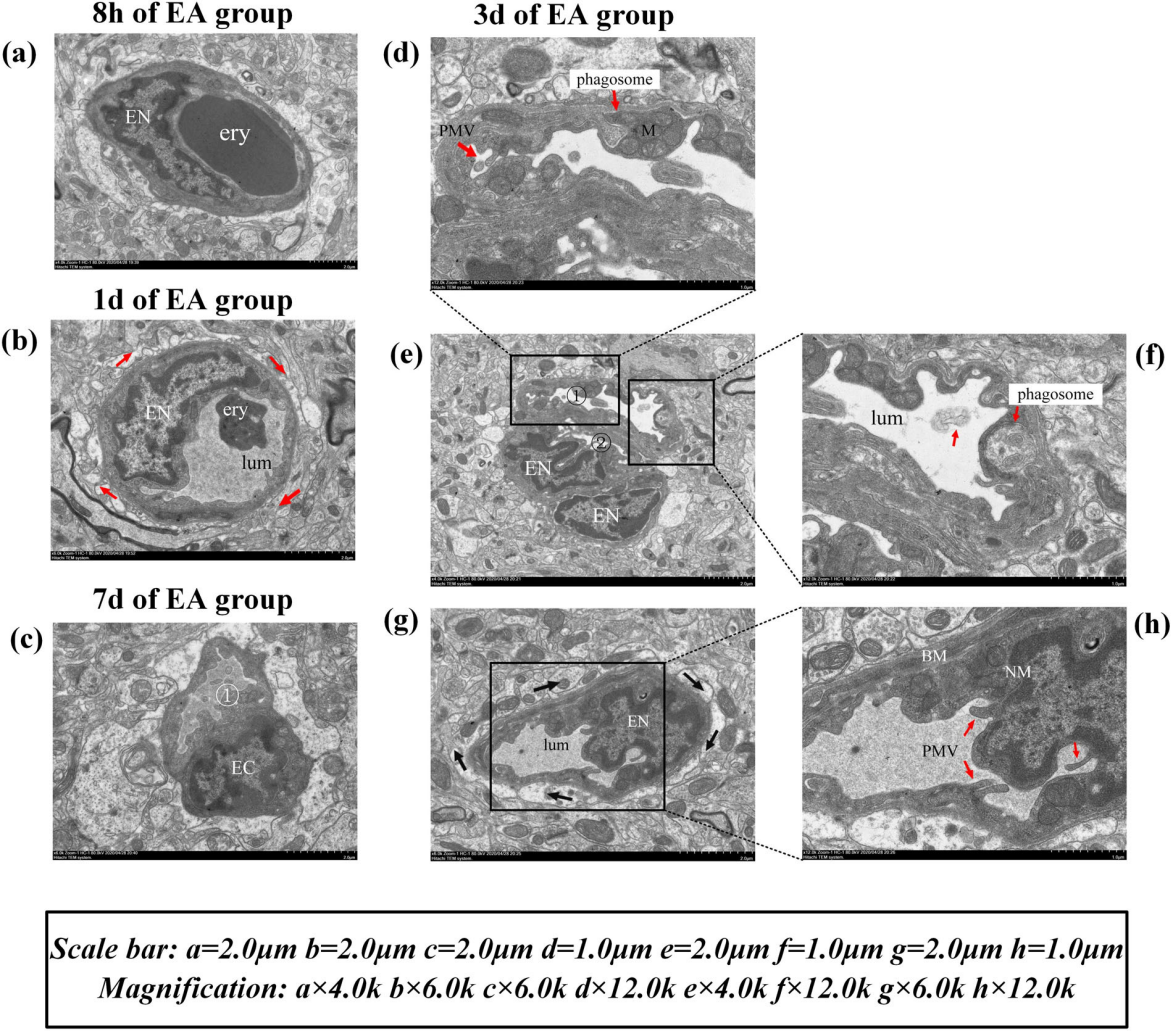
Ultrastructure observation of EA group.

Based on the above findings, we further explored the mechanism of EA treatment. The protein levels of VEGFA, Notch1, and Hes1 were quantified by western blot analysis. The results indicated that VEGFA in the EA group for 8 h and Notch1 in the EA group for 1 day were significantly higher compared with the MCAo/R group at the same time (*p* < .01) (Figure 5a and c), suggesting activation of the VEGF/Notch pathways. Subsequently, the downstream target gene Hes1 protein of the VEGF/Notch pathway is activated. Hes1 in the EA group for 8 h and 1 day were significantly higher compared with the MCAo/R group at the same time (*p* < .01) (Figure 5e). To examine the effect of EA on GV26, SP6, and PC6 to activate the VEGF/Notch pathway, we observed the ultrastructure of the left cerebral cortex in MCAo/R rats. The results show that EA intervention 8 h after MCAo/R surgery can effectively alleviate the swelling of ECs and the formation of intracellular vacuoles (Figure 6a) and reduce the volume of cerebral infarction 8 h after MCAo/R surgery (*p* < .01) (Figure 4a and c), suggesting that the application of EA at 8 h and 1 day after focal CIRI can reduce the brain edema and promote the absorption of edema in cerebral infarction tissue. This is related to the activation of the VEGF/Notch pathway by EA and the elevation of VEGFA, Notch1, and Hes1 protein expression in the infarcted brain area of the MCAo/R rats.

### 3.4 EA upregulated VEGFA, Notch1, and Hes1 protein levels, promoted angiogenesis 3 and 7 days after focal CIRI, and inhibited EC apoptosis

In order to clarify the cascade reaction of VEGF‐Notch1‐Hes1 expression caused by EA treatment and the molecular mechanism of the VEGF/Notch signaling pathway involved in regulating angiogenesis, we further detected the expression of the above proteins in the EA group for 3 and 7 days. We found that the VEGFA of the EA group for 3 days and Notch1 of the EA group for 7 days were significantly higher compared with the MCAo/R group at the same time (*p* < .05 and *p* < .01), and Hes1 of the EA group at 3 and 7 days were significantly higher compared with the MCAo/R group at the same time (*p* < .01) (Figure 5a and c). In combination with the electron microscope, it was detected that the morphology of ECs in the EA group at 3 days was normal and the phagosomes were clearing the damaged mitochondria (Figure 6e and d). Microvascular proliferation can be seen under the electron microscope at this stage (two microvessels can be seen in one ultramicrofield) (Figure 6e). The endothelial nuclei migrate and proliferate, and the cytoplasm of the EC fills the whole EC (Figure 6e). Because the activation of the VEGF/Notch pathway after EA treatment causes the upregulation of VEGFA, Notch1, and Hes1 proteins, EA can reduce the accumulation of damaged mitochondria, promote the self‐renewal of damaged brain tissue, and reduce the occurrence of apoptosis. Compared with the MCAo/R group at the same time, the neurological deficit score of rats in the EA group decreased at each time point, especially at 3 days (*p* < .01) (Figure 4b), and the volume of cerebral infarction in the EA group decreased significantly at 3 days (*p* < .01) (Figure 4a and c). The morphology of ECs in the EA group at 7 days of angiogenesis can be seen under the electron microscope: an EC differentiated into a new microvessel (Figure 6c①). The EC and newborn microvessel morphology are healthy (Figure 6c and e). Taken together, because the angiogenesis regulators VEGFA, Notch1, and Hes1 act on the upstream, midstream, and downstream of the VEGF/Notch signaling pathway, respectively, the effect of angiogenesis after focal CIRI was fully demonstrated after EA treatment (Figures 5 and 6).

## 4 DISCUSSION

EA has been reported to be actively involved in various pathophysiological cascades following ischemic stroke, including angiogenesis and neurogenesis (Chavez et al., 2017; Zhao et al., 2015). A combination of related studies suggests that EA may act on ECs and exert a protective effect on the brain by activating the VEGF/Notch signaling pathway (Fu et al., 2021; Lee et al., 2015; Xu et al., 2022; Zhang et al., 2018; Zhao et al., 2015). In this study, we hypothesized that EA can promote angiogenesis in the ischemic area by activating the VEGF/Notch pathway after focal CIRI, thereby reducing brain injury and promoting neurological recovery. What is the specific molecular biological mechanism? In order to study the neurovascular protection mechanism of EA after ischemic stroke, we created a rat model of MCAo/R and administered EA treatment at different times of reperfusion. As we estimated, the enlarged infarct volume and neurological deterioration in rats could be ameliorated by EA. What surprised us even more was that, under the ultrastructural observation of ECs, EA could not only reverse the necrosis and apoptosis of ECs after focal CIRI but also promote EC migration and proliferation. Microangiogenesis was also observed under ultrastructural microscopy. To further corroborate our findings, we also detected proteins in the VEGF/Notch pathway that are closely related to angiogenesis.

Vascular endothelial growth factor A (VEGF‐A) is a central mediator in postischemic proangiogenic molecules in rodent models and humans (Alrafiah et al., 2021). Currently, emerging evidence demonstrates that VEGFA mediates directed angiogenesis in ECs and appears to be one of the most potent mediators in both physiological and pathological angiogenesis (Geiseler & Morland, 2018; Li et al., 2021; Shibuya, 2011). Furthermore, VEGFA‐mediated neurovascular protection and Notch‐1‐triggered antiapoptotic effects in endothelium and other neural cells may synergistically contribute to neurovascular protection during the acute stage of ischemic stroke (Chen et al., 2020; Li et al., 2022). Our findings show that after focal CIRI, an 8‐h EA treatment can begin to mobilize VEGFA protein (Figure 5a). At 8 h, the level of VEGFA protein in the EA group was significantly higher than in the MCAo/R group. (*p* < .01), indicating that an 8‐h EA treatment activates the central mediator of the VEGF/Notch pathway after focal CIRI. One‐day EA treatment after reperfusion, the expression of Notch1 protein increased. Compared with the MCAo/R group, the expression level of Notch1 in the EA group increased significantly (*p* < .01) (Figure 5c), which is consistent with the conclusion of Takeshita et al. (2007) that Notch1 is a downstream protein of VEGF. Under an ultrastructural microscope, the swelling of ECs was reduced after EA treatment for 8 h and 1 day, and the brain tissue and microvessels of the ischemic area were restored to a homeostatic state. The VEGF/Notch signaling pathway may have been activated in this period after EA intervention. Hes1, the downstream target gene of the Notch pathway, was transcribed in the EA group for 8 h, 1 day, 3 days, and 7 days, and the Hes1 protein level of the EA group was significantly higher than that in the MCAo/R group (*p* < .05 or *p* < .01) (Figure 5e). After 3 and 7 days of EA treatment, the levels of VEGFA and Notch1 proteins continued to increase after reperfusion (Figure 5a and c), and EC proliferation and microangiogenesis were observed under ultrastructural microscopy (Figure 6c and e). Thus, consistently, we demonstrated that after focal CIRI, EA promotes the cascade reaction of increased expression of VEGFA‐Notch1‐Hes1, activating the VEGF/Notch signaling pathway and promoting angiogenesis.

Additionally, it was recently found that the Notch signaling pathway is responsible for integrating spatiotemporal information provided by the VEGF cytokine during ordered endothelial sprouting (Gianni‐Barrera et al., 2011). EA may guide this spatiotemporal information to the morphogenetic events of new vascular network sprouting by activating the VEGF/Notch signaling pathway. Our study has confirmed that EA can activate the associated crosstalk of VEGFA and Notch1 for 8 h, 1 day, 3 days, and 7 days of treatment after focal CIRI. Although it has been reported that EA treatment significantly enhances the expression levels of Notch1 and VEGFA after ischemic injury and exerts a repairing effect on cerebral blood vessels and brain tissue (Zhao et al., 2015; Li et al., 2022), whether the antiapoptotic effect to EC under the ultrastructure is related to the activation of the VEGF/Notch pathway by EA remains to be further explored.

Hairy and enhancer of split (Hes1), a basic helixloop‐helix transcription factor, is a downstream effector gene of the Notch receptor. Studies have shown that Hes1 deficiency in human and mouse cell lines induces apoptosis (Takeshita et al., 2007). Furthermore, Hes1 knockdown exacerbates cerebral infarction and neurological deficits after MCAO (Li et al., 2020a, 2020b). Our study found that the transcriptional level of Hes1 was significantly down‐regulated (*p* < .01) in brain tissue after reperfusion for 3 and 7 days, compared with sham surgery (Figure 5e). In addition, ultrastructural analysis showed that ECs showed apoptosis at 3 and 7 days of reperfusion (Figure 3i, k, and m), and the cerebral infarction volume and neurological function scores of MCAo/R rats were also worse than those of the sham surgery group (*p* < .01) (Figure 4a and b). These findings suggest that the decreased protein level of Hes1 after focal CIRI is closely related to EC apoptosis. It provides useful clues for future research into the ability of EA to promote Hes1 protein transcription as well as activate the VEGF/Notch pathway to exert neurovascular protection after focal CIRI.

After EA treatment, VEGFA, Notch1, and Hes1 increased correspondingly at each period after reperfusion (Figure 5). EA group for 3 and 7 days, the structure of EC proliferation and microangiogenesis was observed (Figure 6e and c), and no apoptotic cells were found. The above results suggest that after EA activates the VEGF/Notch pathway, it not only promotes angiogenesis but also reduces the apoptosis of ECs. Compared with the model group, the volume of cerebral infarction decreased at 3 days in the EA group (*p* < .01) (Figure 4a and c), and the neurological function score also significantly improved at 3 days (*p* < .01) (Figure 4b). The above results suggest that there is a close relationship between neurons and ECs in the process of angiogenesis, and angiogenesis often promotes the recovery of neurological function after cerebral ischemia. Moreover, studies report that postischemic angiogenesis follows a temporal course (Bi et al., 2022; Zhang et al., 2009). ECs begin mitosis as early as 1 day after mouse cerebral reperfusion after 30 min of MCAO, and the number of vessels begins to significantly increase on the 3 days before peaking at 7 days after stroke (Yang et al., 2016). In our study, under the ultrastructural microscope, ECs migrated and proliferated at 3 and 7 days after EA intervention, and angiogenesis was observed under the microscope. It is not difficult to discover that EA has the greatest effect on promoting angiogenesis three and 7 days after focal CIRI.

In terms of experimental design, our research follows the design idea of complementing and supporting each other from macro to micro, from morphology to pathology, from ultrastructure to molecular biology. To demonstrate the effective role of EA after CIRI, electron microscope images of ultrastructural analysis were used as the main line, molecular biology‐related proteins were used as important evidence, and cerebral infarction volume and behavioral scores were used as supporting evidence. Not only that, in the next step, we will transform the knowledge of medical biology gained from the laboratory research—that is, the proangiogenic effect of EA—into improved therapeutic measures for patients with focal CIRI. In clinical practice, it is possible to begin EA treatment 8 h after CIRI. Application of EA 3 and 7 days after CIRI may encourage angiogenesis and aid in the better recovery of ischemic stroke patients. After the results of clinical trials are confirmed, the transition from the lab to stroke clinical practice will be realized. This will make it possible for ischemic stroke patients and clinicians to get better treatment and rehabilitation programs.

Several limitations of our research merit discussion. There are various explanations for the mechanism of cell pathological damage and angiogenesis in cerebral ischemia reperfusion at present (Jurcau & Simion, 2021). The occurrence of cell necrosis and apoptosis is the pathological result of the interaction of receptors and signal pathways at the molecular biological level (Saberianpour et al., 2021). Angiogenesis is also a complex and strictly controlled process (Hatakeyama et al., 2020). Both of them play an important role in the process of ischemic stroke. In our study, it has been confirmed that EA can help encourage angiogenesis after CIRI, which is closely related to the activation of the VEGF/Notch signaling pathway. During this process, it can be found from morphological observation that the pathological changes of EC necrosis and apoptosis have decreased, but this is only a speculation based on the ultrastructural analysis and molecular biology levels. The exact impact of EA on brain cell necrosis and apoptosis still needs further evidence from related detection methods.

Another limitation is the time window for EA to promote angiogenesis after CIRI. Since our EA intervention started 90 min after the successful modeling, but the first observation node was set 8 h after the EA intervention, it cannot be proved whether EA can promote angiogenesis at the superearly stage of CIRI. The discovery of this problem can help us find the direction of improvement for the next stage of experimental research. It is critical to investigate the target of angiogenesis intervention in order to improve the clinical efficacy of stroke. After the onset of an ischemic stroke, early intervention with EA for rehabilitation purposes is recommended. However, the timing mechanism of EA regulating angiogenesis and nerve function reconstruction after CIS is relatively complicated. At present, we still need more work to further prove the therapeutic window of EA and promote the timely repair of brain tissue damage and the reconstruction of neurological function in patients with ischemic stroke during clinical treatment.

Finally, I would want to emphasize that the CIRI investigated in this study—a focal one—is fundamentally distinct from a global one. In this study, the MCAo/R model simulated the most common pathological type of ischemic stroke in neurology, while global cerebral ischemia‐reperfusion injury (GCI/R) is commonly seen in emergency medicine, and cardiac arrest (CA) and return of spontaneous circulation (ROSC) are the most common types of GCI/R in experiments in which rats received the 4‐VO model (Wang et al., 2022). In our research, EA plays an effective role in promoting angiogenesis in focal CIRI.

## 5 CONCLUSIONS

In summary, except for demonstrating that EA can activate the VEGF/Notch signaling pathway to promote angiogenesis after focal CIRI, the more important discovery is that EA can reduce the pathological changes of edema, necrosis, and apoptosis by regulating the morphological changes of ECs and promoting the recovery of neurological function after ischemic stroke. This also confirmed our hypothesis that EA promoting angiogenesis after focal CIRI is an important mechanism for ischemic stroke treatment.

## AUTHOR CONTRIBUTIONS

ZWY and HL designed the intellectual content of the original research and designed important concepts. ZWY, HL, WY, SLX, LYB, and LXD performed the experiments and analyzed the data. ZWY, SLX, and HL drafted the article and revised it critically for important intellectual content. WY and LYB coordinated with EA treatment. LXD assisted in literature retrieval and experimental research. All authors reviewed the manuscript and finally approved the version to be published.

## CONFLICT OF INTEREST STATEMENT

The authors declare that the research was conducted in the absence of any commercial or financial relationships that could be construed as a potential conflict of interest.

### ETHICS STATEMENT

All experiments were performed following the National Institutes of Health Laboratory Animal Care and Use Guidelines (NIH Publications No. 80‐23) and were approved by the Animal Ethics Committee of Tianjin University of Traditional Chinese Medicine, China. (approval No.TCM‐LAEC2019018).

### PEER REVIEW

The peer review history for this article is available at https://publons.com/publon/10.1002/brb3.2912.

## Data Availability

The data used to support the findings of this study are available from the corresponding author upon reasonable request.
